# Vaccinating the world against COVID-19 is a no-brainer

**DOI:** 10.1371/journal.pgph.0000427

**Published:** 2022-05-02

**Authors:** Joseph E. Stiglitz

**Affiliations:** School of International and Public Affairs and Columbia Business School, Columbia University, New York, New York, United States of America; PLOS: Public Library of Science, UNITED STATES

There are many lessons to be learnt from COVID-19, in particular as we think about preparedness for the possible next epidemic.

First, two optimistic notes. There are many achievements. The first echoes what was said in the WHO report on COVID-19 research and innovation achievements [1]—the response of the research and scientific communities across the world has been outstanding. The speed of mobilization, the R&D community and the collaborative and high-quality work of thousands of researchers and research institutions was unprecedented.

But we should remind ourselves that it was based on solid foundations of basic research done over years, funded largely by governments [2]. These achievements were not just based on the research of the private sector. Governments enabled the private sector to use the ideas established by that basic research and bring the vaccines to fruition. We can only imagine how bad things might have been had we not done this basic research.

There is a second achievement that I think we should celebrate: bringing the vaccine to market with billions of doses produced in record time. Again, in this achievement, government and collective action across the world played a vital role.

But matching these achievements were some grave disappointments. We saw the ugliness of vaccine nationalism and a broader hoarding of the kinds of products that would protect us against COVID-19, such as personal protective gear and hoarding of tests [3]. There was a kind of broader nationalism of hoarding COVID-19 products. The result was that there were huge inequities—what was referred to as vaccine apartheid, a hoarding of vaccines.

While in the United States the Centers for Disease Control is discussing making a fourth shot available to everybody [4], three shots have already been made available, much of the world, especially low- and middle-income countries (LMICs), hasn’t been vaccinated.

These inequities in access to vaccines has, in my judgment, been very foolish [5]. To echo another refrain of WHO, no one is safe until we are all safe. The health of each of us is at risk. As long as the pandemic continues in any place in the world, there is a risk of mutations, and mutations which might be more contagious, more dangerous and even more vaccine-resistant.

Not only is our health at risk, but our economy is also at risk [6]. The economic losses from the pandemic have been, literally, in the trillions of dollars. And the International Monetary Fund (IMF) estimates that if the pandemic continues, we lose more trillions of dollars [7]. This is an example of what I would call a “no brainer”. The benefit-to-cost ratio of ensuring that everybody in the world has access to the vaccine is enormous. The OECD estimates that it would cost us 50 billion dollars to ensure that everybody is vaccinated [8]. The benefit would be in the trillions of dollars. And yet the richest countries in the world seem paralysed. They can’t provide the small amounts of money that are required to vaccinate the world.

Some countries like Germany continue to oppose the intellectual property waiver (IPR) that has been put forward more than a year and a half ago at the World Trade Organization (WTO). This IPR waiver is not a change in the basic legal framework we already have embodied in international law, the principle of compulsory licenses, and especially so in the context of a pandemic [5]. It is really hard to understand their opposition to the vaccine waiver.

The IPR waiver may not be a panacea, but it would help [5, 9]. There is evidence that LMICs have the capacity to produce the vaccines. There is an enormous amount of incoherence in the arguments put forward by the opponents of the IPR waiver. Many say, for instance, that LMICs and emerging markets don’t have the capacity to make use of the new technologies.

Of course, that is an argument for the transfer of technology. The governments that have provided so much money for the basic research and even bringing the vaccines to market should put pressure on the pharmaceutical companies to engage in this transfer of technology. But if it were true that the countries, emerging markets, LMICs or others, couldn’t avail themselves of intellectual property, there would be no harm from the vaccine waiver.

I think it’s pretty clear what is really at issue. The real issue is the pharmaceutical companies want to maintain their monopoly profits for as long as possible. If there are a large number of producers of the vaccine, the price will come down and they won’t be enjoying the billions of dollars in profits that they have been enjoying so far. But the profits of pharmaceutical companies are being made at the cost of the lives of thousands and thousands of people, at the cost of enormous inequities. It is also undermining the standing of Western countries that seem to be putting profits over lives [10].

This brings me to the key questions going forward. COVID-19 has exposed and exacerbated the inequalities both within and between countries. It has had disparate effects on the health and the economy of different countries. I’ve written extensively on how LMICs don’t have the resources to resuscitate their economies [6]. The United States, for instance, has spent somewhere around 25% of its gross domestic product (GDP) to maintain the strength of the economy and it has worked [6]. It is one of the reasons why the decrease in GDP in the United States in response to the COVID-19 pandemic has been smaller than other countries, and why we are already not only back to where we were before the pandemic, but we actually have a stronger economy than was anticipated in the months before COVID-19.

But LMICs don’t have these resources. And that’s why some of them have had a decline in their GDP with devastating effects, declining significantly greater than that in the United States and other advanced countries [6].

Well, that’s why it’s all the more important to make sure that LMICs and emerging markets are protected against COVID—and the next pandemic that may be coming down the line.

Given the selfishness of rich nations that’s been exposed, the only way we can be assured that low- and middle-income countries will be protected, the only way that we can make the world safe, given the selfishness, is to have the research and production capacity for making vaccines and other pharmaceutical products distributed throughout the world [5].

Having this production and research capacity distributed throughout the world will enable a quicker and better response to the next pandemic.

What we saw in COVID-19 also showed that there were some further benefits. There would be a faster detection of the disease and new variants and the possibility of a faster response to these new variants. As I think about the priorities and preparing for the next pandemic, surely, ensuring research and production capacity around the world must remain among our highest priorities.
